# Why We Whistleblowers Are Passionate in Our Convictions

**DOI:** 10.1371/journal.pmed.0020281

**Published:** 2005-08-30

**Authors:** Stefan P Kruszewski

**Affiliations:** **1**Harrisburg, PennsylvaniaUnited States of America

Whistleblowers serve no function if they cannot tell their stories. The present story of whistleblowing—as discussed, in part, in *PLoS Medicine*—that involves the pharmaceutical industry, pharmaceutical benefit management corporations, the managed care industry, and the political and lobbying forces that zealously guard their secrets could not have been told without the help of courageous men and women [1, 2] For that reason, those of us who congregated in Washington, D.C., on May 15th, 2005, at the invitation and support of the Public Library of Science and the Government Accountability Project feel particularly humbled and grateful to these two sponsors. Our convictions could not have been aired were it not for the essential First Amendment work of responsible journalists, who exemplify the best in investigatory research.

For me, whistleblowing is not a theoretical exercise. It has a human face and tangible features. It is the face of children and adults who have been injured or killed by misrepresented pharmaceuticals; clinical research trial results that have been sequestered from the scientific community and whose incomplete findings cause injury; and pharmaceuticals that are detailed to physicians, not to save lives or necessarily improve the health or welfare of the recipients, but to make money.

In the lonely and, at times, discouraging world of whistleblowing, we whistleblowers are passionate, and often successful, because our efforts have a different goal than the corporations and political interests whose operations we occasionally challenge. Our goal is to tell the truth. That honest effort is the source of any ethical difference we can or might make. Truth is the basis for the power of a whistleblower, one that can withstand the assault of unprecedented odds against being heard put forth by that sum of political power, expediency, and money.

A whistleblower's success depends upon competent and articulate media. The debate to improve the status quo—be it in pharmaceutical marketing or managed-care decision making—cannot proceed or flourish without it.

Ralph Waldo Emerson, American essayist and philosopher (1803–1882), commented about success (I have adapted his comments for all of us who gathered in Washington in mid-May 2005): “To leave the world a bit better, whether by a healthy child, a garden patch or a redeemed social condition; to know even one life breathed easier because you have lived; this is to have succeeded [as a whistleblower].”
